# Predicting Early Warning Signs of Psychotic Relapse From Passive Sensing Data: An Approach Using Encoder-Decoder Neural Networks

**DOI:** 10.2196/19962

**Published:** 2020-08-31

**Authors:** Daniel A Adler, Dror Ben-Zeev, Vincent W-S Tseng, John M Kane, Rachel Brian, Andrew T Campbell, Marta Hauser, Emily A Scherer, Tanzeem Choudhury

**Affiliations:** 1 Cornell Tech New York, NY United States; 2 BRiTE Center Psychiatry and Behavioral Sciences University of Washington Seattle, WA United States; 3 Department of Psychiatry The Donald and Barbara Zucker School of Medicine at Hofstra/Northwell Hempstead, NY United States; 4 Dartmouth College, Computer Science Hanover, NH United States; 5 Vanguard Research Group Glen Oaks, NY United States; 6 Biomedical Data Science Department Dartmouth Geisel School of Medicine Hanover, NH United States

**Keywords:** psychotic disorders, schizophrenia, mHealth, mental health, mobile health, smartphone applications, machine learning, passive sensing, digital biomarkers, digital phenotyping, artificial intelligence, deep learning, mobile phone

## Abstract

**Background:**

Schizophrenia spectrum disorders (SSDs) are chronic conditions, but the severity of symptomatic experiences and functional impairments vacillate over the course of illness. Developing unobtrusive remote monitoring systems to detect early warning signs of impending symptomatic relapses would allow clinicians to intervene before the patient’s condition worsens.

**Objective:**

In this study, we aim to create the first models, exclusively using passive sensing data from a smartphone, to predict behavioral anomalies that could indicate early warning signs of a psychotic relapse.

**Methods:**

Data used to train and test the models were collected during the CrossCheck study. Hourly features derived from smartphone passive sensing data were extracted from 60 patients with SSDs (42 nonrelapse and 18 relapse >1 time throughout the study) and used to train models and test performance. We trained 2 types of encoder-decoder neural network models and a clustering-based local outlier factor model to predict behavioral anomalies that occurred within the 30-day period before a participant's date of relapse (the near relapse period). Models were trained to recreate participant behavior on days of relative health (DRH, outside of the near relapse period), following which a threshold to the recreation error was applied to predict anomalies. The neural network model architecture and the percentage of relapse participant data used to train all models were varied.

**Results:**

A total of 20,137 days of collected data were analyzed, with 726 days of data (0.037%) within any 30-day near relapse period. The best performing model used a fully connected neural network autoencoder architecture and achieved a median sensitivity of 0.25 (IQR 0.15-1.00) and specificity of 0.88 (IQR 0.14-0.96; a median 108% increase in behavioral anomalies near relapse). We conducted a post hoc analysis using the best performing model to identify behavioral features that had a medium-to-large effect (Cohen *d*>0.5) in distinguishing anomalies near relapse from DRH among 4 participants who relapsed multiple times throughout the study. Qualitative validation using clinical notes collected during the original CrossCheck study showed that the identified features from our analysis were presented to clinicians during relapse events.

**Conclusions:**

Our proposed method predicted a higher rate of anomalies in patients with SSDs within the 30-day near relapse period and can be used to uncover individual-level behaviors that change before relapse. This approach will enable technologists and clinicians to build unobtrusive digital mental health tools that can predict incipient relapse in SSDs.

## Introduction

### Background

Schizophrenia spectrum disorders (SSDs) are complex chronic conditions characterized by a diverse set of symptoms that present themselves heterogeneously throughout the affected population. Symptoms are typically categorized into 2 groups: *positive* symptoms, which are an exaggeration of normal function (eg, hallucinations, disorganized speech or thought) and *negative* symptoms, described as a loss of normal function (eg, lack of expressiveness, apathy, and asociality) [1]. Symptom exacerbation in SSDs leads to a psychotic relapse. Relapse has serious potential consequences, jeopardizing many aspects of patients’ lives, including personal relationships and employment, with an increased risk of patients causing harm to themselves or others [2]. Previous research has estimated that the annual direct medical cost of schizophrenia amounts to US $37.7 billion within the United States, with an even larger indirect cost (US $117.3 billion) [3]. Early detection of relapse could inform time-sensitive clinical efforts that may reduce the severity of relapses or prevent their occurrence altogether.

The heterogeneity of symptoms and the timing of symptom exacerbation make detecting early warning signs of relapse difficult. Relapse symptoms, unlike common first-episode psychosis symptoms, can appear abruptly [2]. In-depth interviews with patients with SSDs describing their prerelapse symptoms show that symptom manifestation is extremely idiosyncratic but often consistent within individuals. Each individual may have their own unique *relapse signature*, and identifying this signature could be the most effective manner of detecting incipient relapse [4]. Traditional measures of relapse come from clinician-administered rating scales that attempt to quantify a patient’s current experience with an SSD [5,6]. However, it is often unlikely that patients present themselves to a clinician when their symptoms begin to re-emerge or worsen, particularly in an illness characterized by cognitive disorganization, loss of insight, and inconsistent treatment delivery systems where it can be difficult to access care [7]. To prevent symptom exacerbation, tools need to be developed that are able to detect early warning signs of relapse outside of the clinic.

Over the past decade, improvements in sensing technologies within smartphones, wearables, and other devices have created new opportunities for remote measurement of mental health symptoms [8,9]. Behavioral data collected with passive sensors from smartphones offer unobtrusive methods to measure trajectories of mental health and mental illness [10-13]. Smartphones can track a diverse set of behaviors and are owned and utilized by most individuals with SSDs [14,15].

The CrossCheck system was the first smartphone-based tool designed to collect passive sensing data as a method of tracking the symptoms of SSDs. The system combines passive sensing with triweekly self-reported survey measures [16]. Using CrossCheck, researchers were able to predict patient self-reported ecological momentary assessments (EMAs) from passive sensing data and combine both the passive sensing and self-reported data to predict clinician-administered Brief Psychiatric Rating Scale (BPRS) scores [17,18]. In addition, researchers were able to detect significant changes in patient smartphone social behavior during the 30 days preceding relapse [19]. Although these analyses provide a foundation for identifying symptom changes that contribute to relapse, it is an open question whether we can predict specific time points of symptom exacerbation that show a clear relapse signature.

Relapse is a rare event, and lack of available data near relapse can make prediction problematic [20]. Anomaly detection is a branch of data mining specifically for the prediction of peculiar, infrequent events [21,22]. Traditional approaches for anomaly detection within time series involve forecasting and use statistical measures based on cumulative sums, moving averages, and regression models that rely on predicting changes in the underlying distribution of the time series [22]. Forecasting human behavior is an extremely difficult problem, and behavioral data from patients with schizophrenia do not traditionally follow the circadian rhythms seen within a healthy population [23,24]. Algorithms designed to learn complex features within time series data are likely to have more success in finding anomalies and detecting behaviors associated with relapse.

More novel approaches to time series anomaly detection use encoder-decoder neural network models to identify anomalies in multivariate time series data. These algorithms have had success in learning complex features, specifically in highly irregular sensing data [25-27]. Unlike statistical approaches, neural networks do not require assumptions about the underlying distribution of the data and are often ideal compared with classical machine learning techniques because they can provide accurate predictions without the need for complex feature engineering. However, there is a tradeoff. It can be difficult to interpret the reasoning behind why neural networks make specific predictions, leading to the common description that neural networks are *black box* models. In medicine, specifically, interpretability is important because clinicians need to justify the risk of using new approaches; thus, it is challenging to introduce neural network–based decision making into the clinical workflow [28]. Machine learning researchers focused on model interpretability have offered approaches to analyze models *post hoc*, after model training, to uncover the relationships between the input features to the network and the network prediction [29]. To successfully implement a neural network–based anomaly detection system within behavioral health, one needs to not only show good results in detection but also provide a process for uncovering the underlying behaviors that lead to an anomaly and provide a clinical translation for those behaviors.

### Related Work

Researchers have begun to utilize anomaly detection to predict early warning sides of psychosis. A pilot study using a combination of mobile sensing features and self-reported survey responses was able to identify an increase in anomalies within 2 weeks of relapse in a small patient population [30]. Another recent study utilized retrospective features extracted from Facebook to create a classifier for detecting the 1-month period before relapse and then analyzed the behaviors that were significantly different within this period [31]. Developing an algorithm to predict specific days of symptom exacerbation before relapse using exclusively passive sensing data could provide clinicians an unobtrusive method to measure SSD symptoms without the need for patient self-reporting.

### Contributions

This study makes the following contributions:

1. We created a variety of encoder-decoder neural network–based anomaly detection models to predict early warning signs of psychotic relapse using passive sensing data collected from a smartphone. To the best of our knowledge, these are the first models designed to predict early warning signs of relapse using exclusively passive sensing data.
2. We provided a post hoc analysis for clinical interpretation of the detected anomalies within the context of SSDs and demonstrated that our algorithm can detect participant-specific relapse signatures.
3. We analyzed how variations in participant data can change model performance to provide guidance for future researchers in digital mental health for model and study design.

## Methods

### CrossCheck System and Study

The CrossCheck system was an Android app combined with a cloud-based data collection and storage platform. The app continuously collected users' passive sensing data and prompted participants every 2 to 3 days to self-report EMAs to track both positive and negative symptoms of SSDs [17,32]. EMAs were not utilized in our anomaly detection system owing to low completion rates across relapse participants. Table 1 provides an overview of the raw passive sensing data collected using CrossCheck. Sensors also collected environmental data, including ambient sound and light. The ambient sound was utilized by the app to classify when conversations occurred near the participant, but the raw sound and light data were not used in this research. Refer to our previous work for more specific information about the data collected during this study [16,17].

**Table 1:** Summary of passive sensing behavioral data collected throughout the study.

Behavior	Description	Derived hourly features
Acceleration	3-axis acceleration data were collected from a smartphone, sampled from 50-100 Hz. Previous CrossCheck studies utilized the Android activity recognition API^a^, which classifies activity data as follows: on bicycle, still, in vehicle, tilting, or unknown. In this study, we chose to use raw acceleration features to make our anomaly detection system independent of a specific activity recognition API platform	Mean acceleration over the hour
App use	CrossCheck recorded apps running on a user’s smartphone every 15 min	Number of unique apps opened within an hour
Call	Phone calls can indicate social interaction and communication. We tracked when incoming, outgoing, missed, rejected, and blocked calls occurred	Number and duration of incoming, outgoing, missed, rejected, and blocked calls
Conversation	Previous studies have investigated the link between conversations, human voice, and mental health [12,33,34]. We detected human voices and conversational episodes using algorithms from our previous work [35]	Number and duration of conversations
Location	Previous research has shown that location can be associated with mental health [12,13,36]. We tracked location information from users through their smartphones	Time in primary, secondary, and all other locations as well as total distance travelled in the hour
Screen activity	The amount of time users spend on their phones can be tracked to learn normal daily behaviors. The time users’ screens were on versus off was recorded	Number of times the phone was used as well as the duration of use
Sleep	On each day, the sleep duration, onset, and wake time were detected. These calculations occurred using a combination of information based upon users' screen time, physical activity, ambient sound, and light [12,37]	Sleep duration, onset. and wake time. As we estimated only the longest sleep episode per day, this is technically a daily feature. We replicated these features across all hours within a single day
Text	Text messages are another indicator of social interaction. We tracked when texts were received, sent, drafted, left in a user's outbox, failed to send, and were queued for sending	Number of received, sent, drafted, outbox, failed to send, and queued messages

^a^API: application programming interface.

The CrossCheck study was a randomized controlled trial (RCT) aimed at testing the efficacy of using passive sensing and self-reported data to identify digital indicators of relapse. The participants enrolled were randomized either into a smartphone arm for passive sensing data collection or into a control arm to receive treatment as usual. In this work, because our goal was to predict early warning signs of relapse from collected passive sensing data, we focused exclusively on the smartphone arm. Participants enrolled in the study were given an Android smartphone for 12 months and instructed to carry the device with them and complete the EMA. Trained clinical assessors met with participants to conduct a baseline assessment of symptoms and functioning. Clinical assessors also conducted follow-up assessments with participants during months 3, 6, 9, and 12 of the study to administer the 7-item BPRS, which measures psychiatric symptoms associated with SSDs [6,16]. Participants’ electronic medical records (EMRs) were also made available to the clinical assessors. The following events, either reported during assessment or recorded within the EMR, were designated as relapse: psychiatric hospitalization, a significant increase in psychiatric care (including more intensive or frequent services, increased medication dosage, or additional medication prescribed) coupled with an increase of 25% from the baseline total BPRS score, suicidal or homicidal ideation with clinical relevance, self-injury, or violent behavior resulting in harm to another person or property [19]. The date of relapse, any notes surrounding the relapse event, and the reason for designating the event as a relapse were recorded. When corroborating evidence surrounding the relapse was not available within the EMR, clinicians worked with participants during the assessments to gain more information regarding the relapse event.

Relapse is an acute event, but when the early warning signs of relapse begin to surface is an open question. Consistent with previous research on early warning signs of relapse, we defined the 30-day period before relapse as the *30-day near relapse period* (NR30), and all data outside of this period were considered *days of relative health* (DRH) [19,31,38].

### Study Protocol

The CrossCheck study was approved by the Committee for Protection of Human Subjects at the Dartmouth College and the Institutional Review Board of the Northwell Health System. The study was registered as a clinical trial (NCT01952041).

### Participants

Participants were recruited into the RCT from several treatment programs at a psychiatric hospital in New York. Participants were recruited through flyers posted at the study site with the research coordinator’s phone number. In addition, researchers reviewed the hospital’s EMRs to identify potential participants. A potential participant’s clinician was contacted by the investigative team, and after describing the study to the patient, clinicians referred patients interested in the study to the research team.

Eligible participants met the following inclusion criteria: (1) a chart diagnosis of schizophrenia, schizoaffective disorder, or psychosis not otherwise specified, (2) 18 years of age, and (3) an inpatient psychiatric hospitalization, daytime psychiatric hospitalization, outpatient crisis management, or short-term psychiatric hospital emergency room visit within 12 months before beginning the study. Individuals were excluded if they had the following: (1) hearing, vision, or motor impairment that would impede smartphone usage (determined using a smartphone demonstration during screening), (2) a below sixth grade reading level (determined using the Wide Range Achievement Test–4th Edition), and (3) unable to provide informed consent (using a competency screener) [16,39].

A total of 1367 individuals were initially assessed for eligibility and 149 were enrolled in the study. Eligible individuals who did not enroll were no longer receiving care at the hospital (n=682), failed to meet the diagnostic criteria (n=131), did not want to participate (n=129), or did not meet the severity criteria (n=108). Of the 149 individuals enrolled, 62 were randomized into the smartphone arm of the study [19]. Participants included in this work (n=60) were required to have had at least 10 DRHs collected by the smartphone app.

### Feature Extraction and Data Cleaning

An advantage of using neural networks for machine learning is that they have the ability to learn intricate features from raw data [40]. We sought to create features for our learning algorithm that were close to the raw data to exploit this fact. Hourly features were created from the raw sensor data. A summary of the hourly features used can be found in Table 1. In addition to the passive sensing features, we included the day of the week and the hour of the day as features in our model. The few features that require more complex calculations are described below.

Android phones track acceleration within a 3D *x, y, and z* coordinate system. This produces 3 values for every acceleration reading, namely *a=(a_x_, a_y,_ a_z_).* We computed the mean hourly acceleration by taking the vector norm of each *a* within a specific hour and averaging over the values.

We also tracked the longitude and latitude locations over time for each participant. The locations for each participant were clustered using the density-based spatial clustering of applications with noise (DBSCAN) algorithm, implemented in the scikit-learn library [41,42]. DBSCAN clusters samples of high density together, requires a minimum number of samples per cluster, and requires a maximum distance, ε, between points to be specified as hyperparameters. We required a minimum of 10 samples per cluster and set ε*=*1 km. For each participant, the 2 majority clusters were tagged as the participant's *primary* and *secondary* locations, and all other data points were grouped together into a third cluster. Finally, we calculated the distance between each pair of longitude and latitude coordinates using the Haversine formula [43]. We then summed the distances over each hour.

Two types of missing data were identified. The first type of missing data (type 1) occurred when there was a sensor reading during an hour for one feature but there was no reading within the same hour for another feature. We imputed missing data for type 1 values with a “0,” indicating our belief that the CrossCheck system was functioning during these hours, but an individual did not partake in specific behaviors that the system records (eg, no texts were recorded within an hour). A second type of missing data (type 2) was identified during hours where all features were missing. We imputed features for the second type of missing data utilizing the mean value of a given feature for that hour. Location features (time spent in primary, secondary, and other locations) were filled differently. We assumed that the participant remained at their last recorded location and filled the features accordingly.

We assumed that by using mean filling for type 2 missing values, we would direct our anomaly detection models to focus on finding anomalies within the actual passive sensing data. That being said, missing values, specifically type 2 missing values, could have an implication for function. For example, if a participant stopped using their phone and the smartphone app, missing values could be an indication of asocial behavior, which may precede relapse. We added an additional feature to the model that indicated the percentage of features filled within a given hour. If this feature was <1, the hour was filled using the type 1 missing data procedure, but if the feature was equal to 1, the hour was filled using the type 2 missing data procedure.

### Encoder-Decoder Models

We developed multiple algorithms to detect early warning signs of relapse using passive sensing data. Patients with SSDs are known to not experience normal circadian rhythms that are typically found within a healthy population [23,24]. Thus, we chose to apply a neural network approach to this problem that has been used for multivariate anomaly detection in irregular sensor data [25-27]. Specifically, we created a fully connected neural network autoencoder (FNN AD) model and a gated recurrent unit sequence-to-sequence (GRU Seq2Seq) model that learned to reconstruct an input time series [44]. A GRU network was used over a vanilla recurrent neural network (RNN) and other popular RNN architectures, such as a long short-term memory (LSTM) network, as the GRUs counter the vanishing gradient problem that occurs when training the vanilla RNNs, and they converge faster during training than LSTM networks [45]. After training the encoder-decoder models, our algorithm learned participant-specific anomaly thresholds based on the model reconstruction error. We discuss the architecture of the encoder-decoder models in this section and describe the thresholding procedure in the subsequent sections.

We considered each participant’s data to be a time series of varying length *L, X={x^(1)^,…,x^(L)^}*, where each *x^(i)^* is a multivariate data point, *x^(i)^∈R^m^*. In our case, each *x^(i)^* represented a set of hourly features for a single participant. We created subsequences of data of length *l* starting at each *i, i={i,…,L-l+1}*. Note that a given data point, *x^(i)^*, could be potentially included within each of the 1,…,*L* subsequences. For the FNN AD model, we let *l=*1, and for the GRU Seq2Seq model, we let *l=*24.

**Figure 1:**
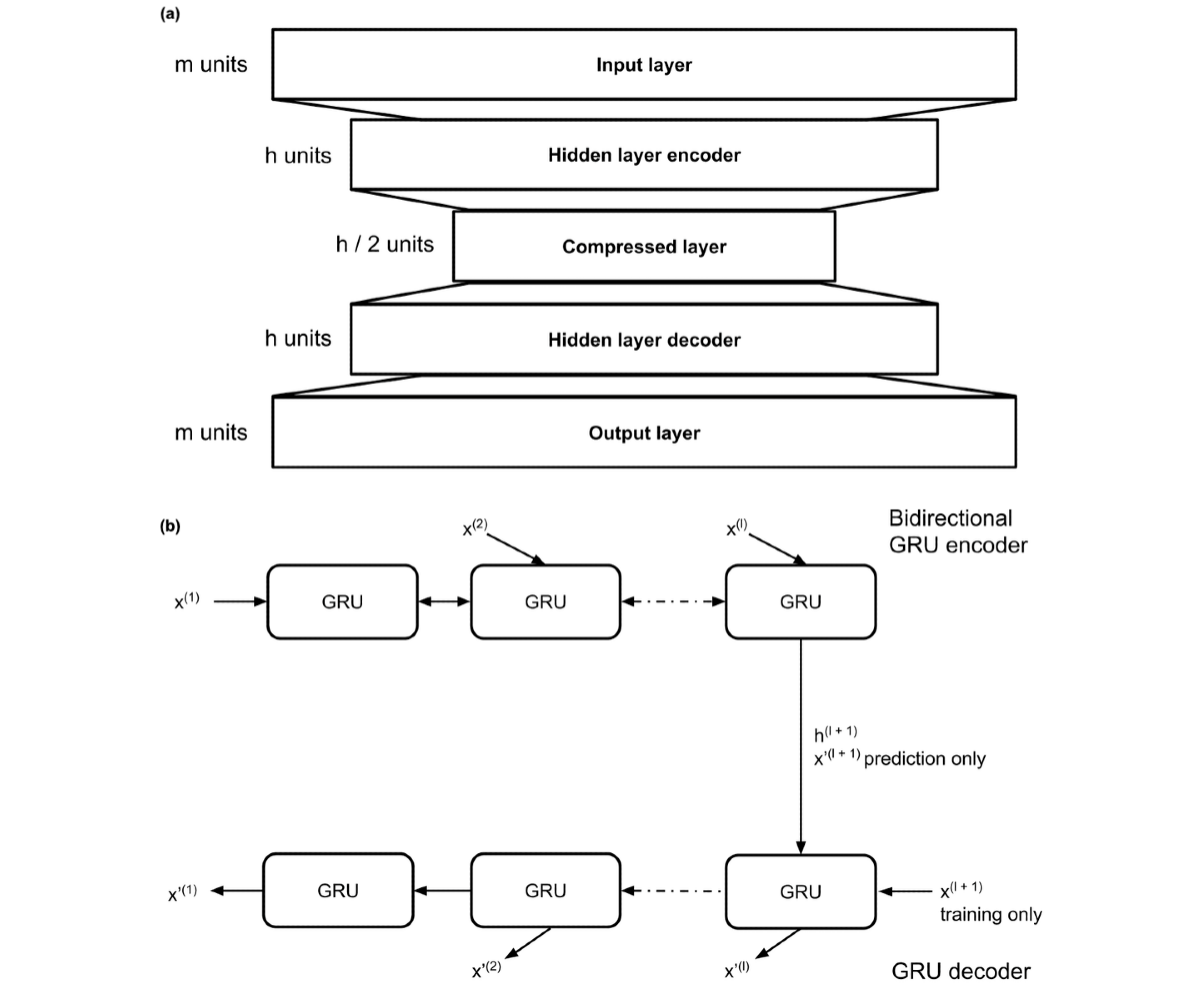
Encoder-decoder neural network architectures. (a) the architecture for the fully connected neural network autoencoder (FNN AD) model. (b) the architecture for the GRU sequence-to-sequence (GRU Seq2Seq) model.

The models were constructed as follows. This is also detailed in Figure 1. The FNN AD model comprised 2 fully connected encoder and decoder layers that compressed an input subsequence to a lower dimension and then recreated the initial subsequence. For the GRU Seq2Seq model, we first input a subsequence of data into a single *encoding* layer of a bidirectional GRU with a specified hidden unit size. A bidirectional layer was used for the encoder because previous research has shown that bidirectional layers improve the results over unidirectional layers [46]. The last cell in the encoding layer outputs a prediction for the next timestep, *x’^(l+1)^*, and encodes hidden information from the entire sequence, *h^(l+1)^*. We then passed this information as inputs into a unidirectional GRU *decoder* layer that reconstructed the subsequence in reverse order: *{x’^(l)^,…,x’^(1)^}*.

### Model Training Procedure

We utilized a similar data-splitting and cross-validation procedure as described in previous work [25-27]. The data for each participant were first split into equal length nonoverlapping subsequences, and the subsequences were placed into 1 of 4 data sets. Defining NR30 as the 30-day near relapse period and DRH as days of relative health (ie, all days not in NR30), the data were split into the following:

1. A training data set, comprising *only* DRH, called *H_R_*. These training data are utilized to train each model.
2. A cross-validation data set, comprising *only* DRH, called *H_CV_*. These cross-validation data are utilized to validate the ability of the models to reconstruct sequences of new data.
3. A cross-validation data set, comprising DRH and NR30, called *N_CV_*. These cross-validation data are used to tune the anomaly detection component of our algorithm as described in the following section.
4. A test data set, comprising *both* DRH and NR30, called *N_T_*. The test data set are used to report the metrics of the anomaly detection algorithm described in the Results section.

We also experimented with the percentage of relapse participant data to include in each of these 4 data sets. Specifically, we experimented with placing different percentages of DRH within *H_R_* and *H_CV_*. We experimented with placing 0%, 20%, 40%, 60%, and 80% of relapse participants’ DRH into *H_R_* and *H_CV_*. DRH for both relapse and nonrelapse participants were split such that 80% of DRH were placed into *H_R_* and 20% into *H_CV_*. Nonrelapse participant data were split entirely between *H_R_* and *H_CV_.*

Monte Carlo cross-validation was used to examine the robustness of the algorithm across different potential *N_CV_* and *N_T_*. We stratified each Monte Carlo sample to place equal amounts of NR30 data per participant within *N_CV_* and *N_T_*. The Monte Carlo procedure was repeated over 100 iterations and the median and IQR of the true-positive rate (TPR or sensitivity), and false-positive rate (FPR) of the current Monte Carlo test set *N_T_* were recorded.

### Anomaly Detection System

We used the trained encoder-decoder models to reconstruct *H_CV_*, *N_CV,_* and *N_T_*, producing *H’_CV_*, *N’_CV_*, and *N’_T_*. For a data point *x^(i)^* in each of these data sets and its reconstructed counterpart *x’^(i)^,* we calculated the absolute error of the data points: *e^(i)^=*| *x^(i)^- x’^(i)^*|.

Within our algorithm, the full-time series was split into subsequences of length *l*, and any point *x^(i)^* could appear in at most *l* different subsequences. Thus, for a point *x^(i)^*, there can exist *l* different predictions, *{x_1_’^(i)^,…,x_l_’^(i)^}.* We filtered our data set to include only points that were predicted *l* times. The error vectors for these data were considered to be normally distributed, *e^(i)^~N(μ,Σ),* and the error between *H_CV_* and *H’_CV_* was used to approximate *(μ,Σ )* parameterizing the *expected* error of our algorithm. We then calculated an anomaly score *s^(i)^∈R* for error vectors between *N_CV_*, *N’_CV_*, and *N_T_*, *N’_T_* using the Mahalanobis distance, which calculates the distance of a point to a distribution as follows: *s^(i)^*=*((e^(i)^*-*μ)^T^ Σ^-1^(e^(i)^*-*μ))^1/2^* [47].

The average anomaly score for a single day was calculated from the hourly scores, *s^d^*. The Mahalanobis distance from data in *N_CV_* was used to optimize an anomaly threshold, τ, for each participant over all *s^d^* for that participant. A day was tagged as an anomaly if *s^d^>τ* or normal if *s^d^≤τ*.τ was chosen to maximize the ratio between the TPR divided by the FPR, or TPR/FPR, defining a *true positive* as an anomaly detected within NR30 and a *false positive* as an anomaly detected on a DRH. Optimizing this ratio maximized the number of anomalies detected during the NR30 when minimizing the number of anomalies detected during DRH. This τ was applied to the Mahalanobis distances from the held-out test sample, *N_T_*, and the final results using the best τ for each participant's *N_T_* were recorded.

### Evaluation Metrics

We used the TPR/FPR ratio as an evaluation metric to rank model performance. By maximizing this ratio, we subsequently maximized the sensitivity and specificity of our models. Sensitivity and specificity are metrics commonly used within medicine to assess the strength of a diagnostic test [48]. The sensitivity is equivalent to the TPR and the specificity is equivalent to the true negative rate (or 1–FPR). Thus, by maximizing the TPR/FPR, we found a model that maximized both sensitivity and specificity.

Anomalies are rare events; thus, it is unlikely that every day within NR30 would contain an anomaly. Clinically, we assumed that an anomaly detection system for early warning signs of relapse would be relevant as long as the anomalies were rare (low sensitivity and high specificity), but increased (TPR/FPR>1) within NR30. This increased signal could then be used to find passive sensing features that distinguished anomalies within NR30 from anomalies identified within DRH.

### Baseline Model and Evaluation

We used a k-nearest neighbors local outlier factor (LOF) model as a baseline comparison against our neural network models [49]. The LOF model estimated the local density around each data point using a k-nearest neighbor algorithm and then compared the local density of a given data point with the local density of its neighbors. If the point was in a substantially less dense area, it had a higher calculated LOF. We initially fit an LOF model for each relapse participant utilizing *H_R_* with the number of neighbors equal to 10, and we incremented the number of neighbors by 1 until the mean and SD of the LOF scores under *H_CV_* converged. We could then utilize the approach described above to calculate anomalies by considering the distribution of LOF scores obtained under *H_CV_* and learning an appropriate anomaly threshold for *N_CV_*. The LOF model was trained and tested using scikit-learn [42].

Neural network models were created using TensorFlow and Keras libraries [50,51]. Models were trained until the validation loss from *H_CV_* converged. We used cross-validation for all neural network models to determine the optimal hidden layer size (between 10 and 50 units), the percentage of DRH from relapse participant data to include within *H_R_* and *H_CV_* (between 0% and 80%), and the τ that maximized the TPR/FPR ratio on *N_CV_* (between 0 and 20). For the LOF model, we also optimized the number of neighbors utilized for the local density within each relapse patient.

We applied 2 forms of regularization to train the neural networks. For both the GRU Seq2Seq and the FNN AD models, we used early stopping to terminate model training when the reconstruction error from *H_CV_* increased. In addition, we applied dropout (rate=0.2) and recurrent dropout (rate=0.2) to the GRU Seq2Seq model. Dropout masks, or *drops*, inputs randomly within the network, whereas recurrent dropout adds this mask between the recurrent layers at each timestep [52]. This exposed the trained network to different permutations of the training data to prevent overfitting. Batch normalization was briefly used during model creation, but we found that batch normalization did not improve anomaly detection performance and was not used to train the final iteration of the models.

## Results

### Data Overview

We collected a total of 20,137 days of mobile sensing data from 60 patients with SSDs. Relapse events were recorded for 18 of 60 participants (30%) during the 1-year study, totaling 726 days of data collected within any NR30 data (0.037% of the total days of data collected). Table 2 provides a summary of the data collected from the relapse and nonrelapse groups.

**Table 2:** Summarized data characteristics for relapse and nonrelapse participants (continuous characteristics listed by median [IQR]).

Characteristics		Relapse		Nonrelapse	
Patients, n		18		42	
Age at beginning of study (years), median (IQR)		33 (23-47)		40 (26-50)	
Female, n (%)		8 (44)		17 (40)	
Number of days of data collected per participant, median (IQR)		335 (285-346)		295 (176-361)	
**Missing hours of data (type 2), median (IQR)**					
	Number of hours		2309 (1333-2551)		1785 (660-2871)
	Percentage of total hours		25.73 (14.77-28.73)		27.17 (7.72-52.50)
**Diagnosis, n (%)**					
	Schizophrenia		9 (50)		17 (40)
	Schizoaffective disorder		7 (39)		18 (43)
	Psychosis NOS^a^		2 (11)		7 (17)
**Assessment at baseline, median (IQR)**					
	BPRS^b^ (7-item) total		29 (23-33)		24 (21-29)
**Lifetime hospitalizations, n (%)**					
	1-5		13 (72)		30 (71)
	6-10		1 (6)		8 (19)
	11-15		1 (6)		3 (7)
	16-20		1 (6)		0 (0)
	>20		1 (6)		1 (2)
	Missing or declined		1 (6)		0 (0)
**Distribution of relapse events, n (%)**					
	1 relapse event		14 (78)		N/A^c^
	2 relapse events		1 (5)		N/A
	3 relapse events		3 (17)		N/A

^a^NOS: not otherwise specified.

^b^BPRS: Brief Psychiatric Rating Scale.

^c^N/A: not applicable.

### Anomalies Increased Near Relapse

**Figure 2:**
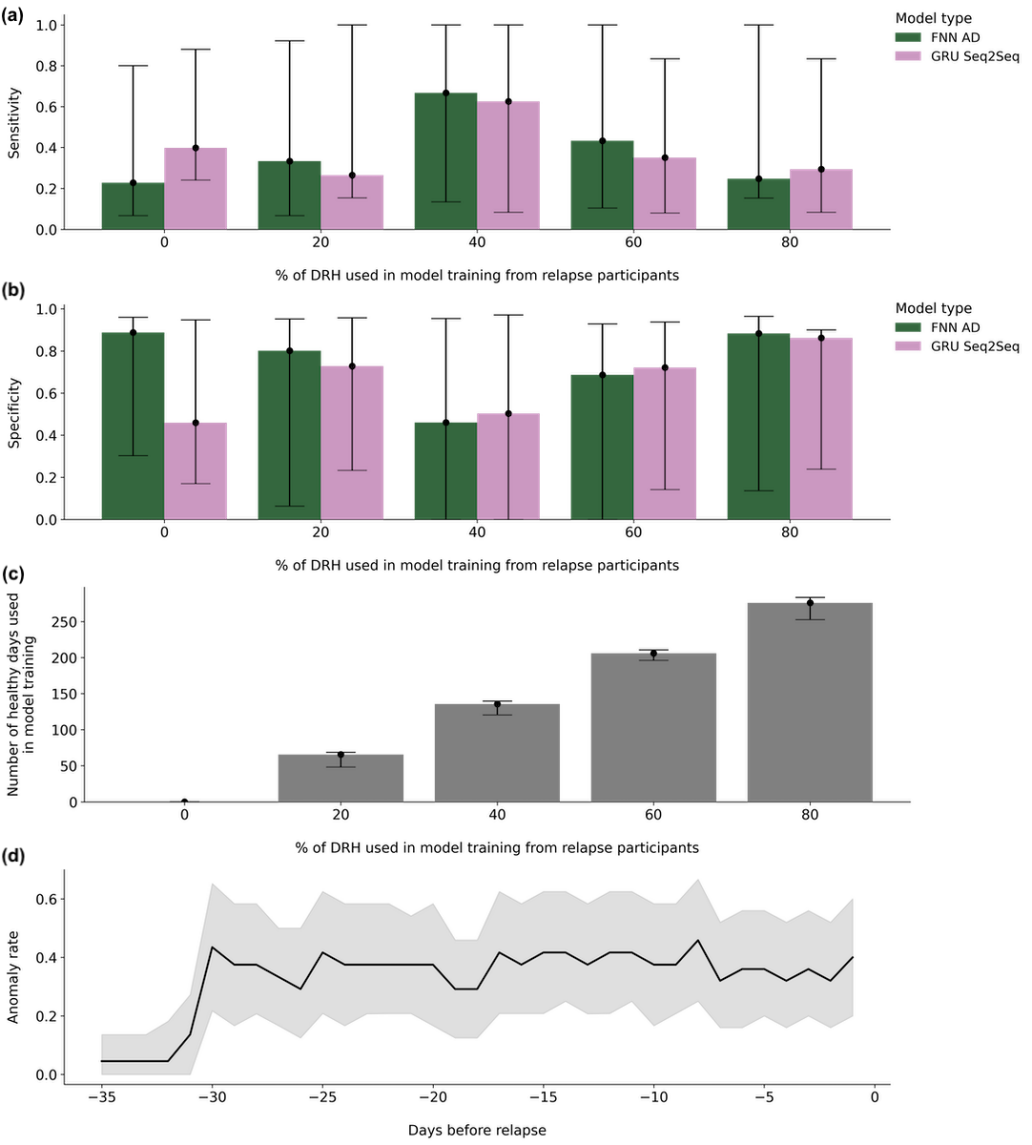
Overall model results, the anomaly rate of the best performing model across the near relapse (NR30) period and in (a-c) split by the DRH used in model training. In (a-c), the bar heights describe the median value of the metric listed on the y-axis across study participants and the error bars show lower and upper quartile values (25% and 75% percentiles of the data). In (a) and (b), local outlier factor (LOF) models are not shown as they did not hold predictive power. (a) Sensitivity, or true positive rate, of the models and (b) specificity, or true negative rate. (c) Median number of DRH used to train each model from each study participant. (d) Average (blue line) and 95% CI (gray shading) anomaly rate across relapse participants beginning 35 days before relapse using the best performing model (fully connected neural network autoencoder, 80% of DRH in train, 40 hidden units). DRH: days of relative health.

The highest performing cross-validation results for each model, with hyperparameters, are shown in Table 3. All results are listed using median (IQR) sensitivity and specificity. Across all model architectures, the FNN AD model using 80% of the data from DRH with 40 hidden units had the highest rank across participants (9.28), achieving a median sensitivity of 0.25 (IQR 0.15-1.00) and specificity of 0.88 (IQR 0.14-0.96). LOF models did not show predictive power (sensitivity 1.0 and specificity 0.0) and were not included in our results. Figure 2 shows the resulting sensitivity and specificity achieved from models trained on different percentages of DRH. Adding a larger percentage of DRH to model training initially increased the sensitivity and decreased the model specificity, but then decreased the sensitivity and increased the specificity as more data were added. Figure 2 shows that the anomaly rate increased before the NR30 period but then remained fairly constant among participants.

**Table 3:** Cross-validation results per model type within relapse participants listed by median (IQR).

Model	Rank	Days of relative health in train, %	Hidden units	Sensitivity, median (IQR)	Specificity, median (IQR)
FNN AD^a^	9.28	80	40	0.25 (0.15-1.00)	0.88 (0.14-0.96)
GRU Seq2Seq^b^	12.72	80	50	0.29 (0.08-0.83)	0.86 (0.24-0.90)

^a^FNN AD: fully connected neural network autoencoder.

^b^GRU Seq2Seq: gated recurrent unit sequence-to-sequence.

### Anomaly Detection System Identified Specific Near Relapse Behaviors

**Figure 3:**
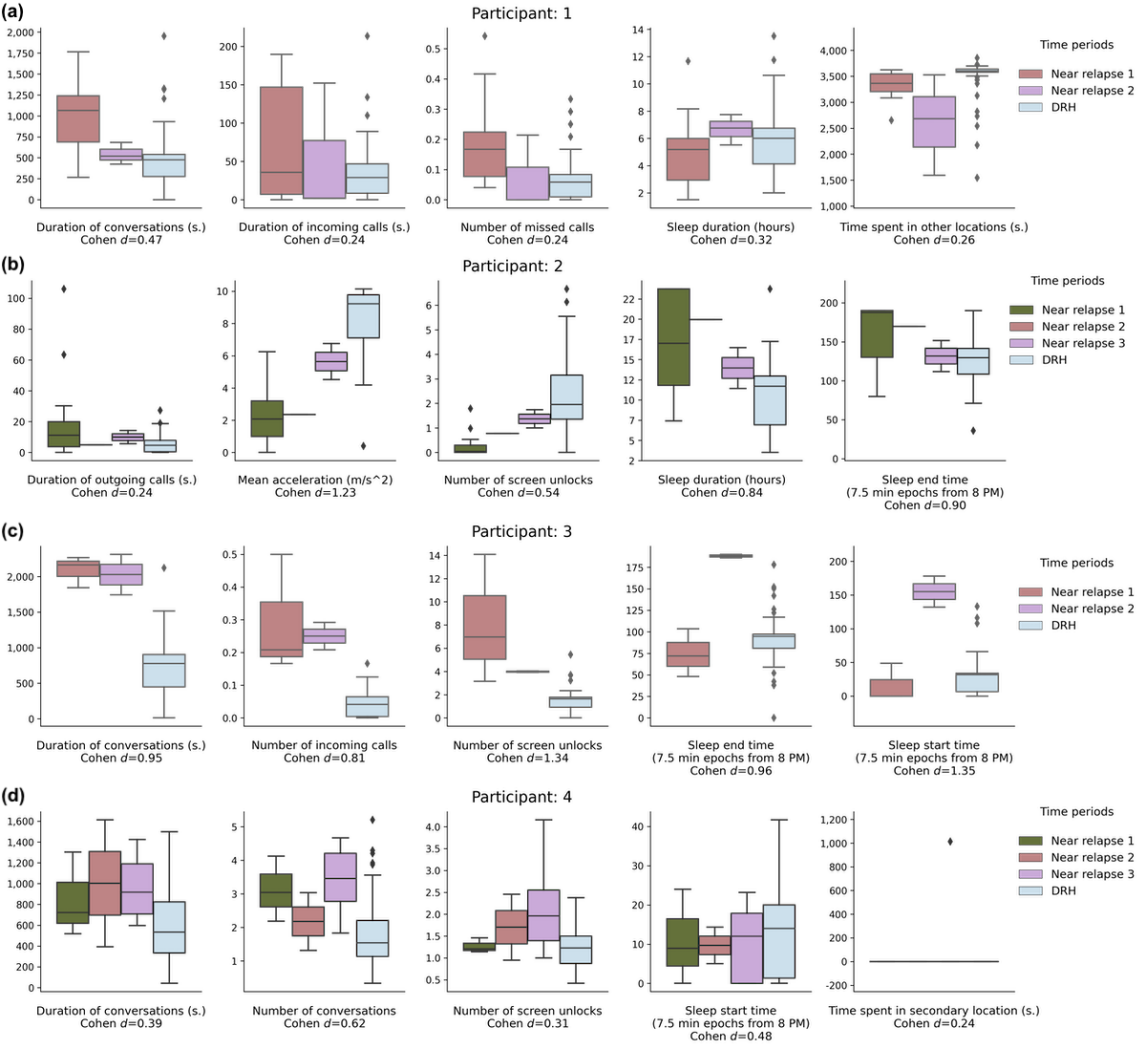
The hourly features that had the greatest effect on differentiating identified anomalous days near relapse (NR30) from all DRH within the 4 multirelapse participants. We used the Cohen d to identify the 5 features that were the most differentiated. Each subfigure, (a-d), displays boxplots comparing the distribution of these features on anomalous days within each NR30 period compared with all DRH. The center line in the boxplot is the median value, the box limits are the IQR, and the whiskers are 1.5 x the IQR. Points outside of the whiskers are greater than or less than 1.5 x the IQR. A lower IQR signifies that the median result is more generalizable. For example, in (a), we identified anomalies within 2 NR30 periods, described in the figure as Near relapse 1 and Near relapse 2. The 2 left boxes on each plot show the distribution of the feature for anomalies detected within each of these 2 NR30 periods and the right box shows the distribution of this feature on all DRH outside of the 2 NR30 periods. NR30: 30-day near relapse period. DRH: days of relative health.

Previous research has shown that individuals often report symptom exacerbation, which could be used to predict the onset of relapse [4]. Identifying participant-specific behaviors that are consistent during relapse would give clinicians a potential signature to identify when a patient needs clinical support. A total of 4 participants within our study relapsed multiple times. We performed a post hoc analysis using our best-performing algorithm across participants (FNN AD, 80% of DRH in train, hidden unit size=40) to compare features on NR30 anomalous days with DRH within multirelapse participants. We used Cohen *d* to calculate the effect of continuous features on discriminating an NR30 anomaly to any DRH and the OR for calculating whether type 2 missing data appeared more frequently in NR30 anomalies [53]. Figure 3 shows the distribution of the 5 features with the largest effect per participant on differentiating detected anomalies within NR30 from DRH.

Notes surrounding each relapse, extracted from the participant’s EMR or obtained during clinical visits, were compiled by a team of trained clinical assessors. We used these notes as a qualitative validation to understand whether the identified features from our analysis were presented to clinicians. We now briefly describe the results of each comparison for features that were identified to have a large effect (Cohen *d*>0.8), medium effect (0.5<Cohen *d*≤0.8), or the feature with the largest effect if no features with a large or medium effect were identified [54]. The ORs indicated that type 2 missing data did not discriminate anomalies within NR30 from DRH for multirelapse participants (OR<1 for all multirelapse participants). The features are described in detail in the Methods section.

#### Multirelapse Participant 1

We did not identify a feature with a large or medium effect for this participant. The conversation duration had the largest effect (Cohen *d*=0.47), which increased before relapse, as shown in Figure 3. Clinical notes from the first relapse event indicate that the participant was hospitalized because she was tired of hearing voices, which suggested that her neighbors were constantly talking about her. Notes from the second relapse did not describe any participant behavior.

#### Multirelapse Participant 2

The participant’s mean acceleration (Cohen *d*=1.23), sleep end time (Cohen *d*=0.90), and sleep duration (Cohen *d*=0.84) had a large effect. Figure 3 shows that the mean acceleration decreased in all 3 NR30 periods for this participant, whereas the sleep duration and end time increased. Clinical notes from the first relapse identified that the participant had been feeling ill, specifically that his “brain was shaking.” On the second relapse, the participant stated that he felt like he “was going to die,” and was feeling depressed. The clinician wrote that the patient “has had difficulty sleeping.” Notes regarding the third relapse indicate that the participant had been disorganized, physically aggressive toward his mother, and was barely sleeping.

#### Multirelapse Participant 3

The participant’s sleep start time (Cohen *d*=1.35), number of smartphone screen unlocks (Cohen *d*=1.34), sleep end time (Cohen *d*=0.96), duration of conversations (Cohen *d*=0.95), and number of incoming calls (Cohen *d*=0.81) had a large effect. Figure 3 shows abnormal behavior in the sleep start and end times for all relapse periods, but is inconsistent in the direction of how the behavior differs from the median value across each relapse. The number of screen unlocks, incoming calls, and duration of conversations increased in both relapse periods. Notes regarding the first relapse did not identify any specific behavioral changes. Clinical notes from the second relapse identified that the participant had been spending his days “making music and beats” and was sleeping less at night, but had increased sleep during the day. The notes also identified the participant as having auditory hallucinations.

#### Multirelapse Participant 4

One feature, the number of conversations, had a medium effect (Cohen *d*=0.62) for this participant. Figure 3 shows that the number of conversations increased during all 3 relapse periods. Notes from the first relapse did not describe any specific behavioral differences in the participant. Clinical notes from the second relapse indicated that the participant presented herself to outpatient psychiatry with “signs of catatonia” and that the participant had mostly stopped speaking, although she had occasional spontaneous speech. We were not able to obtain notes regarding the third relapse event.

### Anomalies Contained Fewer Hours of Type 2 Missing Data

We found that type 2 missing data did not have an effect on distinguishing anomalies within NR30 for the 4 multirelapse participants. We wanted to examine this question more broadly to determine how missing data influenced all detected anomalies. We conducted a one-sided Mann-Whitney *U* test to test the following hypothesis: predicted anomalies contain a smaller number of type 2 hours filled compared with all other days. Individual participant factors were controlled for using participant-specific Mahalanobis distance thresholds for anomaly designation. Anomalies had a median of 0 (IQR 0-6) hours of data filled using the type 2 missing data procedure, and all other days had a median of 2 (IQR 0-16) type 2 hours of data filled. The one-sided test was significant (*U*=514,546; *P*<.001), indicating that anomalous days were significantly less likely to contain type 2 missing data.

### Variations in Relapse Participant Data Affected the Quality of Anomaly Detection

We analyzed the participant-level anomaly detection results to determine how variations in data quality affect the generalizability of our model. Table 4 summarizes the results of using linear regression to assess the significance between the sensitivity and the specificity of the highest performing model (FNN AD, 80% of DRH in train, hidden unit size=40) and the data quality parameters. All data quality parameters were significant (*P*<.001). Increasing the number of days of raw data and the percentage of days within NR30 increased the sensitivity of the model (ß=.60, 95% CI 0.48 to 0.72; ß=.73, 95% CI 0.49 to 0.97) but decreased the specificity of the model (ß=−.69, 95% CI −0.81 to −0.57; ß=−.71, 95% CI −0.95 to −0.47). Increasing the number of days per NR30 period and the number of relapse events decreased the sensitivity of the model (ß=−.43, 95% CI −0.52 to −0.34; ß=−.82, 95% CI −1.02 to −0.62) but increased the specificity of the model (ß=.33, 95% CI 0.23 to 0.43; ß=.87, 95% CI 0.67 to 1.07).

**Table 4:** Linear regression results between sensitivity and specificity and different data parameters.

Parameters	Sensitivity			Specificity	
	Coefficient β	*P* value	Coefficient β		*P* value
Days of raw data	.60 (95% CI 0.48 to 0.72)	<.001	−.69 (95% CI −0.81 to −0.57)		<.001
Days per near relapse period	−.43 (95% CI −0.52 to −0.34)	<.001	.33 (95% CI 0.23 to 0.43)		<.001
Percentage of days near relapse	.73 (95% CI 0.49 to 0.97)	<.001	−.71 (95% CI −0.95 to −0.47)		<.001
Relapse events	−.82 (95% CI −1.02 to −0.62)	<.001	.87 (95% CI 0.67 to 1.07)		<.001
Intercept	.00 (95% CI −0.04 to 0.04)	>.99	.00 (95% CI −0.04 to 0.04)		>.99

## Discussion

### Principal Findings

In this study, we created the first model, exclusively using passive sensing data from a smartphone, to predict behavioral anomalies that could indicate early warning signs of psychotic relapse. Developing an anomaly detection system from exclusively passive sensing data requires minimal effort for data collection from the participant and could lead to more objective and unobtrusive ways of monitoring symptoms of SSDs. Our anomaly detection system achieved a median sensitivity of 0.25 (IQR 0.15-1.00) and specificity of 0.88 (IQR 0.14-0.96; a 108% increase in anomalies near relapse), indicating that anomalies increased before relapse but were restricted to specific days within the defined NR30 period. Once we identified anomalous days within NR30, we demonstrated that our methodology can be used to identify participant-specific behavioral signatures that occur across multiple NR30. In the future, anomaly detection models could be used to identify days that contain these signatures and supervised learning approaches could then be deployed to detect these signals as early warning signs of relapse. Identifying patient-specific behaviors that change exclusively before relapse could provide clinicians an indicator to measure when patients are declining in health and create time for early intervention.

Anomalies increased within NR30, but with low sensitivity. We believe this low sensitivity could be owing to our choice of an NR30 period or our decision to use the TPR/FPR ratio as a validation metric for this work. We chose an NR30 period because past research has shown that early warning signs of relapse might begin to develop up to 1 month before the actual relapse event [19,31,38]. Our low sensitivity indicated that only specific days within this 30-day period were considered anomalies, and training algorithms in the future to target these specific days could increase sensitivity. Another approach to increase sensitivity would be to shorten the number of days included in the near relapse period. For example, previous work using a combination of smartphone social behavior and self-reported EMAs to detect anomalies before relapse identified a 14-day near relapse period [30]. We observed an increased anomaly rate 30 days before relapse (Figure 2), which remained fairly constant; therefore, we did not further investigate shortening the near relapse period. It is important to note that the algorithm we used may result in a constant anomaly rate during a near relapse period of any prespecified length as these algorithms are trained to look specifically for behavioral differences within these periods.

In addition, we used the TPR/FPR ratio for model selection rather than directly optimizing for sensitivity or specificity. Most machine learning algorithms use the area under the receiver operating curve to assess the predictive power of a model, which we did not feel was appropriate for our anomaly detection algorithm. Anomaly detection, by definition, searches for extremely rare events. To introduce this process into a clinical workflow, we would need to strike a balance between highlighting potential early warning signs of relapse without overburdening the healthcare system with a high anomaly rate. We felt this could be achieved using our modeling approach as we showed an increase in anomalies before relapse without sacrificing the specificity of our results.

To increase model sensitivity in this context, more clarity is needed on what should constitute behaviors that can be used to identify early warning signs of psychotic relapse. A process can then be created where we first use anomaly detection to identify candidate relapse signatures and then train supervised learning algorithms to identify these signatures. This would, in turn, limit the feature space to the behaviors per individual that were differentiated before relapse. In addition, identifying these specific signatures as a starting point for a positive signal would allow us to clarify whether false positives were merely noise or hold clinical significance. In this study, a relapse event was indicated for most of our participants by either a psychiatric hospitalization or a significant increase in symptoms as reported by clinician-administered BPRS. It is possible that symptoms were exacerbated on days outside of NR30, and our system detected these days as anomalies. This symptom exacerbation was not given clinical oversight; thus, we had no way to validate whether these anomalies should be considered true positives.

Days that contained more hours of type 2 missing data, in which no passive sensing data for the entire hour existed, were significantly less likely to be tagged as anomalies. Our approach to using mean filling for type 2 data was based on 2 assumptions: (1) that we would like to prioritize behavioral features collected from passive sensors for anomaly detection and (2) that it is possible that a large quantity of missing data might be a sign of asocial behavior and we should also account for missing data with an additional feature that tracks the amount of data missing over an hour. Previous work on anomaly detection with missing data has analyzed the effects of various data filling methods on anomaly detection results. With the assumption that imputed data points should not be detected as anomalies, the work found that this assumption can hold true if the imputed values are located in high-density regions of the feature distribution [55]. In this work, anomalies were significantly less likely to contain missing values (*P*<.001), indicating that the Mahalanobis distance per individual was less for imputed hours and anomalies were more likely to include non–type 2 data points. With this first assumption in mind, it is possible that the potential effect of missing data on relapse was ignored. For example, the missing data feature did not distinguish anomalies within NR30 in the 4 multirelapse participants. More research needs to be conducted on how different missing data imputation procedures can affect mental health symptom prediction algorithms.

We observed that increasing the amount of relapse participant data used for model training did not always increase the resulting sensitivity and specificity. Figure 2 shows that our model performance increased in sensitivity and decreased in specificity when we increased the percentage of DRH from relapse patients used in model training from 0% to 40%. We then observed a reverse trend (decreased sensitivity, increased specificity) when we increased the amount of DRH from 40% to 80%. This demonstrates that as models learned participant-level behaviors, there was a threshold for the amount of data required for model training (approximately 135 days from data from Figure 2) before a model could begin to distinguish anomalous behaviors within NR30. We also found that our anomaly detection system is sensitive to the quality of the relapse participant data. Table 4 demonstrates that increasing the total percentage of NR30 days increased the sensitivity (ß=.73), but not if this increased the average number of days within NR30 (ß=−.43), and increased the total number of relapse events (ß=−.82). Subsequently, having a higher number of relapse events increased (ß=.87) the specificity of the model, but not if this increased the percentage of days within NR30 (ß=−.71), and increased the number of days of raw data (ß=−.69). Taken together, our results depended on identifying homogeneous behavioral signals that occurred exclusively in NR30.

Given the importance of finding homogeneity in the signal, we examined whether we could uncover consistent signals in participants that might indicate SSD symptom exacerbation. It can be difficult to introduce neural network models within clinical practice owing to their *black box* nature, even though they can achieve higher performance than classical machine learning models [28]. In our work, interpretability is critical because clinicians need to develop an understanding and trust of an algorithm's decision-making process. We utilized a post hoc notion of interpretability to identify participant-specific features that differed during NR30 anomalies [29]. We chose the effect size, a metric traditionally used to measure the strength of a treatment in an RCT, to identify the most differentiated features within NR30 [54]. We identified features with a medium to large effect (Cohen *d*>0.5) in 3 of 4 multirelapse patients. The features we identified encompass different aspects of social behavior, sleep, and physical activity.

We searched the literature to interpret the behavior changes that we identified within NR30. Our previous work identified that smartphone social behavior decreased across participants before relapse [19]. We examined smartphone social behavior at the individual level. Multirelapse participant 3 increased their smartphone social behavior before relapse, but, from contextual notes, we discovered that this participant experienced auditory hallucinations, potentially explaining the increased conversation duration detected by the smartphone as well as other increased smartphone social behaviors found. Multirelapse participant 4’s number of conversations increased with a medium effect (Cohen *d*=0.62), contrasting the physician’s notes, which stated that the participant was barely speaking and potentially catatonic. Figure 3 shows that the elevated conversation signal, whether from the participant or the environment, was unique to anomalies within each of the 3 NR30 periods.

In addition, we detected that changes in sleeping behavior had a large effect on 2 participants and decreased acceleration had a large effect on 1 participant. Previous research has shown that patients with SSDs are at a significantly higher risk of developing a sleep disorder or worsened sleep near relapse [56,57]. Multirelapse participant 2’s detected sleep duration increased before relapse. In addition, the participant’s acceleration decreased. Social withdrawal and physical inactivity are common symptoms of SSDs. These symptoms interfere with functioning, potentially leading to relapse, and relapse can produce aggression [58,59]. The symptoms identified were consistent with the clinician’s explanation of the second relapse event for this participant, which described changes in sleep and aggressive behavior. Thus, the features that were most differentiated are consistent with past research identifying early warning signs of relapse.

It is important to note that although we found differentiated features for each participant that were consistent with the notes surrounding relapse, the changes detected by the passive sensors were not always consistent with the changes described in the clinical notes. For example, we found that participant 2’s sleep increased before relapse, with a large effect, whereas the clinician’s notes stated that sleep decreased. Similarly, for participant 4, the number of conversations increased before relapse with a medium effect, whereas the clinician’s notes stated that the participant exhibited signs of catatonia. The smartphone algorithms we used to detect conversation relied on ambient sound to detect human voice and conversational exchanges, but do not necessarily detect the voice of the participant [34]. In addition, the sleep algorithm used detected sleep based on a combination of phone usage, ambient light, stationary behavior, and environmental silence, all features that might occur when someone is still but not necessarily sleeping [37]. When interpreting the result of a *black box* algorithm clinically, we need to interpret the algorithm’s results in the context of the technical capabilities of the passive sensing system used before judging the outputs of the system literally. Thus, although smartphones can find meaningful relapse signatures, the interpretations of these signatures should be corroborated with the patient and other qualitative information to better understand the underlying behaviors that preceded relapse.

### Designing a Relapse Prediction System

We hope that this work moves researchers one step closer to creating a clinical intervention system to predict early warning signs of relapse that can be deployed within the clinical workflow. We reviewed digital mental health and mobile health (mHealth) literature to understand how such a system could be deployed. The MONARCA system was created to help individuals with bipolar disorder track disease symptom trajectories using a combination of both passive sensor data and self-assessment [10]. A field trial of the MONARCA system demonstrated the difficulties in obtaining both patient and clinician buy-in when forecasting mental health symptoms, as patients were not convinced of the accuracy of the passive sensing data and clinicians were unsure of steps to take if the system forecasted symptom exacerbation [60]. Although the possibility of a clinician having patient data *at their fingertips* seems appealing, it is also a liability for clinicians if they have 24/7 monitoring capabilities and choose not to act when a patient is potentially in danger [61]. One possible solution to this issue is to introduce a *clinical technology specialist* into a patient’s care team whose responsibility is to successfully introduce and maintain technology-based services within the clinic [62]. It is evident that there is a gap between technology intervention creation and implementation.

Overall, acceptability will continue to play a large role in implementing mHealth interventions. The PD_Manager mHealth platform, a platform created to track symptoms of Parkinson's disease using passive sensing, provides an example of an mHealth tool where researchers specifically tested the acceptability of the platform to patients and clinicians before testing the effectiveness of the system [63]. To help increase the acceptability of mHealth tools, digital mental health and human computer interaction researchers are focused on solving mHealth implementation hurdles using a user-centered design framework, where technology is created and refined in an iterative process that places the proposed interventions directly in the hands of relevant stakeholders [64]. Creating the technology behind a relapse prediction system is a small piece of the puzzle compared with the larger implementation challenges that will be faced when deploying it.

**Figure 4:**
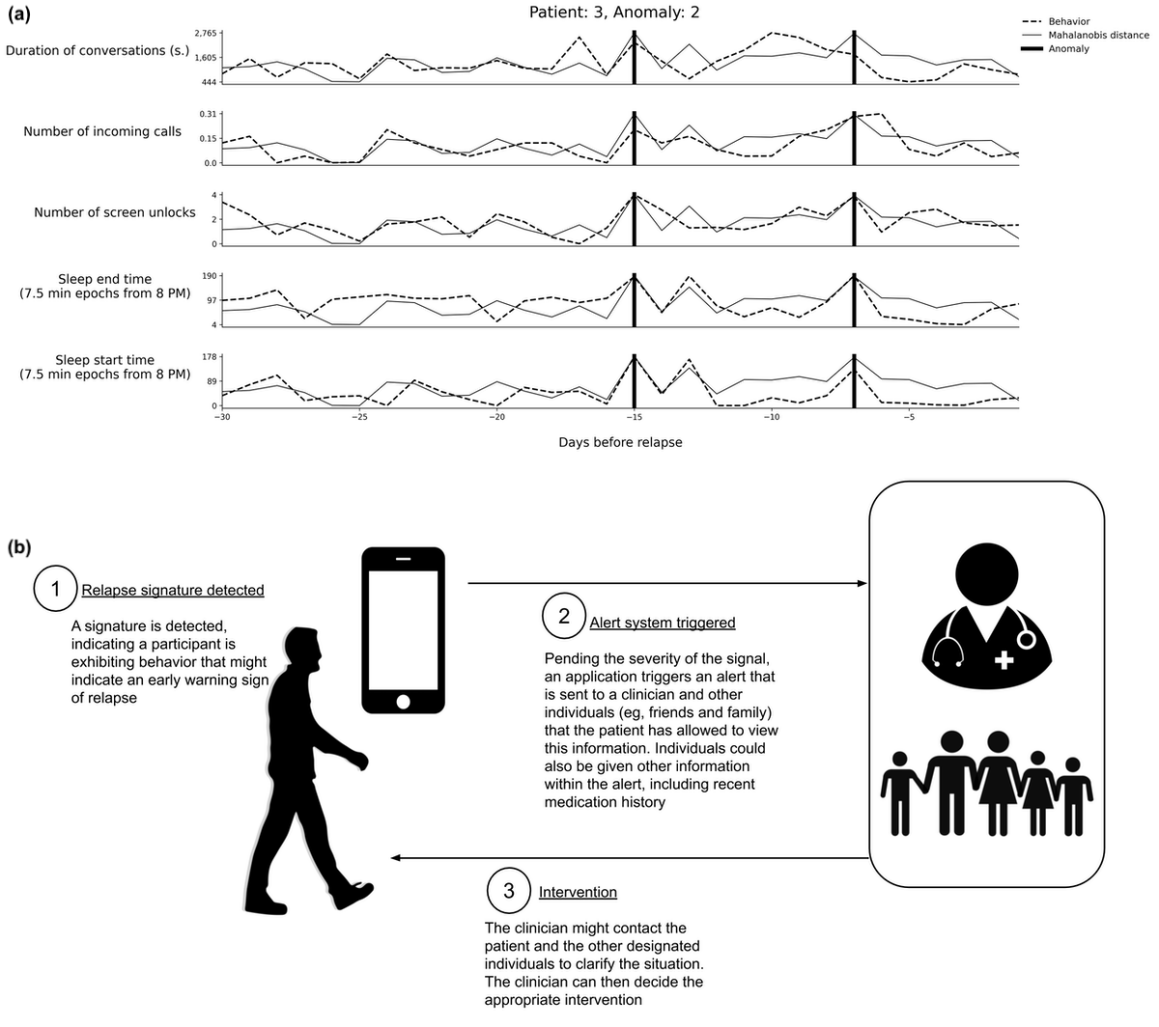
Example of an anomaly visualization and clinical intervention system. The dashed black lines in (a) each represent an hourly feature trajectory from the anomaly detection system, as identified on the y-axis, during a 30-day near relapse period (NR30). The gray line on each plot is the Mahalanobis distance, which can be interpreted as an anomaly score that increases as we are more likely to detect an anomaly. The 2 vertical thick black lines on each plot are detected anomalies. (b) Example of how this information could be utilized by a clinician or other individuals designated by the patient to intervene during symptom exacerbation. The system would be tuned to send alerts only when a patient is in crisis and not overburden the clinician and the healthcare system.

Figure 4 presents an example framework for an SSD behavioral monitoring and intervention system utilizing anomaly detection. Many questions remain regarding implementing this system, including the level of patient interaction with the system, how and when detected anomalies are presented to the clinician or other relevant parties, and a defined procedure that an individual should take to intervene in care when anomalies are detected. The implications of this work can only be truly justified when a system for detecting early warning signs of psychotic relapse has been deployed within the clinical workflow.

### Limitations

The primary limitation of this study was the limited sample size. Our study consisted of 60 participants with SSDs, including 18 participants who relapsed. Most participants did not relapse at multiple points throughout the study and we could not assess whether the features underlying anomalies were consistent with relapse for those participants. To the best of our knowledge, this is still the largest study utilizing anomaly detection to predict early warning signs of relapse exclusively from smartphone behavior.

### Comparison With Previous Work

Previous work using CrossCheck focused on identifying symptom exacerbation by predicting participant responses to the BPRS, a common tool for measuring symptoms of SSDs [6,18]. CrossCheck data have also been used to identify significant associations in smartphone social behavior between the 30-day period before relapse and all other days [19]. We showed that we can predict behavioral anomalies preceding relapse events recorded with and without BPRS. In addition, to the best of our knowledge, only one previous study has utilized anomaly detection to identify early warning of psychotic relapse using a combination of smartphone data and self-reported behaviors [30]. This study used a statistical approach to identify near relapse anomalies within 3 participants. We expand this work by showing that anomaly detection can be used to predict an increase in anomalies before relapse on a larger data set of 18 participants. In addition, we derived features from passive sensing data exclusively, creating an anomaly detection method that does not rely on patient self-report.

### Future Work

Future work should develop approaches to identify early warning signs of relapse across larger and more diverse patient populations with SSDs. These approaches could be tested across different smartphone passive sensing apps such that they become platform independent. In addition, researchers should train models to detect patient-specific relapse signatures, which could increase model sensitivity. Finally, a tool should be codesigned with clinicians and patients for remote monitoring of SSD symptoms.

### Conclusions

In summary, we created an anomaly detection model using encoder-decoder neural networks to predict early warning signs of psychotic relapse. Our model predicted an increase in anomalies within the 30-day period preceding relapse. We developed a methodology to uncover behaviors that change before relapse, which could be used to identify patient-specific relapse signatures. Finally, we discussed the implications of this work and showed an example visualization of a remote monitoring system for SSDs. We hope that this work advances the field of digital mental health to create effective remote monitoring systems for serious mental illness.

## Abbreviations

BPRS: Brief Psychiatric Rating Scale
DBSCAN: density-based spatial clustering of applications with noise
DRH: days of relative health
EMA: ecological momentary assessment
EMR: electronic medical record
FNN AD: fully connected neural network autoencoder
FPR: false-positive rate
GRU: gated recurrent unit
GRU Seq2Seq: gated recurrent unit sequence-to-sequence
LOF: local outlier factor
LSTM: long short-term memory
NR30: 30-day near relapse period
RCT: randomized controlled trial
RNN: recurrent neural network
SSD: schizophrenia-spectrum disorder
TPR: true-positive rate
